# Woodlot management and livelihoods in a tropical conservation landscape

**DOI:** 10.1007/s13280-020-01484-9

**Published:** 2021-02-04

**Authors:** Karen Bailey, Jonathan Salerno, Peter Newton, Robert Bitariho, Shamilah Namusisi, Rogers Tinkasimire, Joel Hartter

**Affiliations:** 1 Environmental Studies Program, Sustainability, Energy and Environment Community, University of Colorado Boulder, 4001 Discovery Drive, Boulder, CO 80303 USA; 2 Department of Human Dimensions of Natural Resources, Colorado State University, Campus Box 1480, Fort Collins, CO 80523 USA; 3 Institute of Tropical Forest Conservation, Mbarara University of Science and Technology, P.O Box 44, Kabale, Uganda; 4 Makerere University, P.O BOX 7062, Kampala, Uganda; 5 Campus Box 1480, Fort Collins, CO 80524 USA; 6 P. O Box 36839, Kampala, Uganda

**Keywords:** Bwindi Impenetrable National Park, Collective action, Livelihoods, Stretcher group, Uganda, Woodlot

## Abstract

In biodiversity hotspots, there is often tension between human needs and conservation, exacerbated when protected areas prevent access to natural resources. Forest-dependent people may compensate for exclusion by managing unprotected forests or cultivating planted woodlots. Outside Bwindi Impenetrable National Park in Uganda, household wood product needs are high and population growth puts pressure on the environment. We investigated the role of privately and collectively managed woodlots in provisioning wood products and supporting local livelihoods. We found that households relied heavily on woodlots for daily needs and as resources during time of need. We also found that locally relevant social institutions, called stretcher groups, played a role in the management of woodlots, providing shared community resources. Privately and collectively owned woodlots support local livelihoods and wood product needs in the region. Long-term management of forests in Uganda should consider the value of woodlots and the mechanisms required to support them.

## Introduction

Tropical forests hold exceptionally high biodiversity value and support the livelihoods of millions of people globally. Locally, forests provide fuelwood, food, building materials, and non-timber forest products (NTFPs), while buffering livelihoods against negative impacts of shocks and disturbances (Kamanga et al. 2009). In addition, forest landscapes maintain ecosystem services supporting much larger human populations than those directly dependent on locally harvested forest products (Lindenmayer and Franklin 2002). However, forest landscapes face increasing threats, including forest product over-harvesting, along with agricultural clearing and a changing climate (Lewis et al. 2015).

To stem the loss of tropical forests and associated biodiversity, protected areas (PAs) are a primary conservation strategy. Twenty-three percent of tropical moist forests are currently under protection and the number of protected areas continues to grow following international commitments to protect biodiversity (Spracklen et al. 2015). Historically, the establishment of strict PAs (e.g., no extractive human activity allowed) has often limited access to natural forests and forest products by adjacent communities (Andrade and Rhodes 2012) and caused eviction and dispossession of local people (West et al. 2006). Such outcomes have often occurred without the provision of land, access to alternative sources of resources, or creation of viable alternative livelihood strategies (West et al. 2006; Lele et al. 2010). Consequently, forest-dependent people lacking access to traditional sources of forest products face a challenge: meeting daily livelihood requirements for fuelwood, building materials, and NTFPs. The areas surrounding PAs thus become the primary source for forest product needs. This intensifies sustainable development challenges in such landscapes, requiring natural resource management outside of PAs to support both livelihoods and conservation goals.

In areas of high human population density, forest-dependent people may compensate for exclusion from forests within PAs by protecting and managing unprotected forests, engaging in agroforestry practices, or cultivating managed woodlots (throughout, we use ‘forests’ to refer to native trees and ‘woodlot’ to refer to cultivated stands or groves of non-native trees). The establishment of managed woodlots is increasingly common among PA-adjacent communities, can support many of the wood product needs provided by forests, namely fuelwood, timber, and NTFPs (L’Roe and Naughton-Treves 2017), and can potentially offset harvest pressure on forests. As a result, in some landscapes, there has been a dramatic shift in land-use management, with many households and communities planting woodlots to meet wood product needs (Rudel 2009). However, it is unclear whether these woodlots can provide all of the necessary resources and income for communities, while supporting their livelihoods and well-being (Ndayambaje and Mohren 2011). If woodlots are unable to meet the wood needs of local communities, people in these landscapes are faced with difficult choices: illegally extracting resources from PAs; becoming increasingly dependent on purchased forest products; sharing resources with family members; receiving donations; or out-migration. Understanding the extent to which smallholder livelihoods are reliant on wood sourced from woodlots and the potential role of woodlots in mitigating these different alternatives is a critical area of research in dynamic tropical forest landscapes (Meyfroidt and Lambin 2011).

A range of management practices can be used to maintain woodlots and forests outside of PAs for local use. Private ownership or freehold tenure systems may effectively maintain these woodlots and forest fragments (Jagger et al. 2005). Alternatively, through formal or informal institutions, community-based forest management can maintain forests that fulfill community forest product needs (Zoysa and Inoue 2016). Additionally, higher order forest governance, such as central state authorities, may significantly shape community autonomy and authority, and ultimately the outcomes for shared forests and impacts on PAs (Nygren 2005). The costs and benefits associated with individual and collective management of unprotected forests vary drastically across contexts and geographies and have significant implications for human well-being and forest conservation (Mendoza and Prabhu 2005). Individually and collectively managed woodlots may contribute to the sustainable provision and equitable use of wood products, while relieving pressure on forests. Alternatively, management and benefits from both private and shared woodlots may be limited to people with power, therefore contributing to inequality of access to forest products (Pasgaard and Chea 2013). Understanding collective and individual woodlot management and potential associated differences in livelihoods will inform a more robust framework within which to understand the role and value of natural resource management strategies (Hartter and Ryan 2010). Disentangling these relationships is especially important in the context of tropical forest landscapes, which maintain relatively high human growth rates and populations particularly susceptible to the impacts of socio-environmental and climatic change (Laurance 2013; Malhi et al. 2014).

This research asks whether and how individual and collective management of planted woodlots outside of protected areas can support livelihood needs in a tropical forested and agricultural landscape. Specifically, we ask:

1. To what degree are smallholders reliant on wood products sourced from private woodlots for their subsistence and/or income-generating livelihoods?
2. Is woodlot ownership associated with differences in wood product use or livelihood measures?
3. Is woodlot management (collective vs. individual) associated with differences in wood product use and livelihood measures?

### Forest management in the Ugandan Albertine Rift

In the Albertine Rift, high rates of species endemism and biodiversity coupled with dense human populations create a biodiversity hotspot and a critical need to understand the use and management of forests and woodlots outside PAs (Myers et al. 2000; Seimon and Plumptre 2012). Much of the region is also characterized by fertile volcanic soils and relatively high rainfall, creating ideal conditions for food crop and tree cultivation and continued migration into the region, putting increasing pressure on limited agricultural land (Gatarabirwa et al. 2000). Increased agricultural needs create challenges associated with soil erosion and degradation, limited fallow land, and less land available per capita (Karamage et al. 2017; Salerno et al. 2018), which limits capacity for livelihood activities, and may increase pressure on PAs and conflict over resources (Tumusiime et al. 2011; Mwesigye and Matsumoto 2016). Woodlots are thought to increase capacity for livelihood activities and, for many of the poorest families in the region, woodlot and forest resources are critical sources of income that reduce wealth inequality, potentially influencing decisions to maintain unprotected forests and/or harvest illegally from protected areas (Olupot et al. 2009; Tumusiime et al. 2011).

**Fig. 1:**
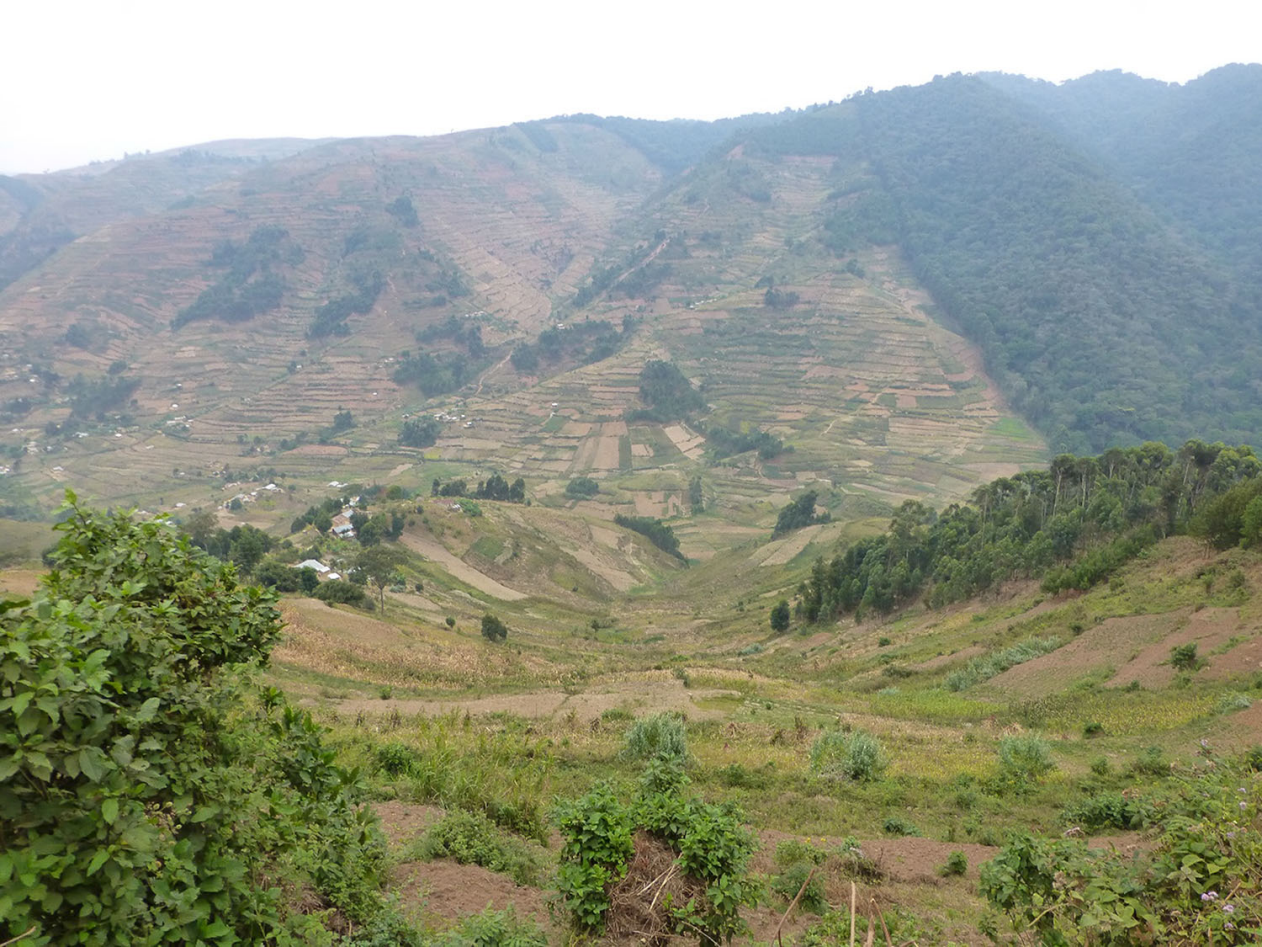
Steep landscape with forest boundary, woodlots, and farmland outside Bwindi Impenetrable National Park, Uganda. Photo by Joel Hartter

The complex land tenure systems in the Albertine Rift also provide an interesting backdrop in which to investigate woodlot and forest management outside PAs. Historically, local Ugandan institutions played important roles in governing and maintaining land tenure systems and managing forests, but a long history of colonial centralization, immigration, and conflict has altered management institutions and local agency over forest management (Banana et al. 2007; Hartter and Ryan 2010). In southwestern Uganda, *ebibiina by’engozi* (also known as *abataka*, ‘stretcher groups,’ from hereon) also provide a potential framework for community collective action (Katabarwa 1999). Stretcher groups are traditional organizations that support access to healthcare and funeral services. Ugandan stretcher groups derive their name from the stretchers used to transport the ill from homes to hospitals and clinics or the deceased to be buried (Cunningham 2014). Due to the steep terrain and separation of villages by steep slopes and deep valleys, travel by foot or via motorbike was, and remains, the most practical means (Fig. 1). These groups emerged to take the sick or injured on a stretcher to the nearest healthcare facility to seek treatment. Stretcher groups continue to do so, serving their original purpose, and have expanded to operate as an organizational level below the official local council to adjudicate conflict, manage local resources, and maintain social networks (Katabarwa 1999; Hamilton et al. 2000). The Ugandan context (i.e., forest-dependent communities, biodiversity hotspot, and stretcher groups as important local institutions) provides an opportunity to study the linkages between forest and woodlot management strategies and livelihood outcomes.

## Materials and methods

### Study area

Our study was conducted in the Ugandan Albertine Rift, a region exemplary of the tradeoffs between conservation goals and immediate human needs that define biodiversity hotspots (Myers et al. 2000). The Albertine Rift is an ideal region to investigate forest and woodlot management outside PAs as it is home to exceptional biodiversity, dense natural-resource-dependent human populations, and a number of ecologically and economically important protected areas. The Albertine Rift is a complex landscape of mountains, wetlands, savanna, and forest, encompassing over 300 000 km^2^ across Uganda, Rwanda, Burundi, the Democratic Republic of the Congo, Zambia, and Tanzania. Our study system was comprised of communities, forests, woodlots, cropland, and pastures adjacent to Bwindi Impenetrable National Park (BINP) in southwest Uganda. Together with the transboundary Virunga Complex, BINP protects the entire population of the world’s remaining endangered mountain gorillas, along with other threatened primates and high altitude endemics in a diverse assemblage of flora and fauna (Plumptre et al. 2007).

**Fig. 2:**
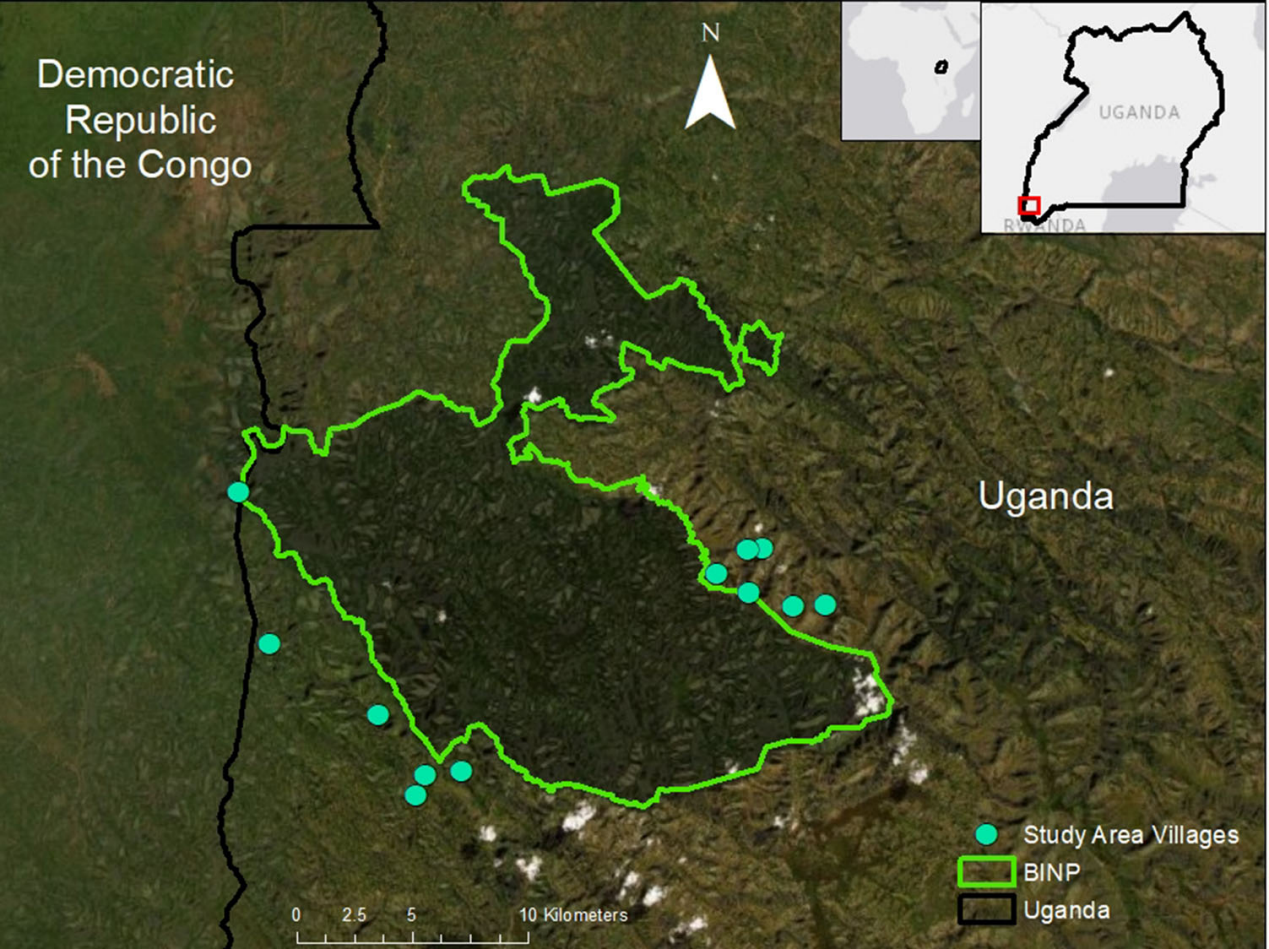
Study area map showing Uganda, southwest Uganda, Bwindi Impenetrable National Park, and villages included in the study

The region where our study was located had the highest human density and population growth of anywhere in the Ugandan Rift (329 people/km^2^; Salerno et al. 2018). We conducted research in twelve villages within two districts, Kisoro and Rubanda, directly adjacent to BINP (Fig. 2). Each of the twelve communities was purposefully selected for representativeness along a range of key factors: elevation, distance to trading center, degree of engagement with organized community forests (i.e., based on non-governmental organizations (NGO) involvement), and agroecological conditions. Additionally, due to the presence of community forests in Kisoro district, villages were paired within Kisoro, such that we selected one village with a community forest and one village without a community forest. The landscapes surrounding these communities included small but intact multiple-use forest and woodlot fragments. Unprotected forests provide critical ecosystem services and a wide range of natural resources that support human livelihoods (Muhwezi et al. 2009; Hartter 2010). In addition, the history of community organization and stretcher group participation provides a strong foundation for collective action for natural resource management (Muhwezi et al. 2009).

### Data collection

We collected data to examine individual and collective action regarding the use and management of forests and woodlots outside PAs using two methodologies: community focus group interviews and quantitative household surveys. All research protocols followed accepted social science research standards (see, e.g., Ibbett and Brittain 2019; Brittain et al. 2020) and were approved by our Institutional Review Board (IRB protocol 14-0145), the Uganda National Council for Science and Technology, and local community leaders and authorities. All protocols included verbal informed consent from all participants prior to research activities and participants were assured confidentiality.

#### Focus group interviews

We conducted 14 focus group interviews, with at least one in each of the 12 villages. The average focus group size was 8.5 people (min = 6, max = 17). Focus group participants were selected with the help of village leaders, with the aim of including a representative diversity of gender, age, and perspective. Focus group participants were not paid, but were compensated for their time with small, locally appropriate gifts after the conclusion of each interview. Group interviews were conducted by the authors and field assistants in Rukiga and English.

The focus group interviews were guided, but open-ended, and were designed to generate contextual information and understanding about community livelihoods, including their resource needs (especially wood product needs), resource utilization (especially of forests and woodlots), and their interactions with the national park. Group interviews covered the following the topics: village demographics, community and household wood product needs, individually and collectively owned woodlots (species composition, size, ecological concerns), individually and collectively owned forests, governance and management of forests and woodlots, characteristics of community organizations, including stretcher groups (forest ownership, membership, size, decision-making), and ongoing challenges associated with meeting wood needs and managing woodlots and forests. The focus group interviews also informed the design and implementation of household surveys, in that these themes were carried forward to household surveys, made locally relevant through interview questions.

#### Household surveys

We conducted 419 household surveys between June 2018 and November 2019. Surveys were conducted with the male and female head of household. Household survey participants were not paid for participation. Surveys were conducted in Rukiga by seven trained enumerators fluent in Rukiga and English and data were recorded on paper and entered into a digital database after each survey was completed. We implemented a balanced randomized sample of ~ 50 households per village. Randomization was conducted at the household level, with households randomly selected from village rosters.

Household surveys were structured around the objectives of the study: to understand how individual and collective management of planted woodlots outside a protected area supports livelihood needs in a tropical forested landscape. Surveys operationalized the interview themes noted above into structured questions. Informed by interviews, surveys included a set of quantitative variables to measure household demographic, physical assets, farming strategies and resources, health and well-being, woodlot and wood product use and access, and stretcher group involvement.

### Analyses

Qualitative data analysis involved working with enumerators to identify recurrent themes, relationships, and patterns related to wood product needs and sources of wood products, private and collective woodlot management, and stretcher group activities. Through content analysis, we then classified and summarized focus group interview data to highlight descriptive results and contextualize quantitative results.

To assess patterns of reliance on woodlots and household livelihood trends, household data on the metrics described above were aggregated and summary statistics were calculated. For all other analyses, we estimated multilevel generalized linear mixed-effect models (Raudenbush and Bryk 2002). To evaluate the relationship between woodlot ownership and household-level livelihood measures, a binary response of woodlot ownership was estimated as a function of household wealth (as measured by the Uganda poverty probability index, which estimates the likelihood of a household falling below the poverty line based on household characteristics (Schreiner 2011)), maximum household education, amount of land owned, and stretcher group membership, with varying intercepts for village (Hedeker 2003). To evaluate the relationship between woodlot ownership and meeting household wood needs, a binary response of household ability to meet wood needs was estimated as a function of woodlot ownership, woodlot size, and duration of woodlot ownership, with household-level control covariates, poverty probability index, maximum household education, and amount of land owned, and varying intercepts for village. To evaluate the relationship between access to collectively owned woodlots and meeting household wood needs, we created a two-way contingency table with two categorical variables: access type (no lot access, group lot access only, private lot access only, access to both a group lot and a private lot) and if a household met their wood needs. We first conducted a *χ*^2^ test of independence to test for a relationship between the two variables (Agretsi 2003). We then fit a log-linear model to the data on woodlot access and wood needs to further assess the relationship between woodlot access type and meeting household wood needs. Log-linear models are commonly used to model associations between categorical variables (Agretsi 2003). Note that “access” here refers to ownership of private woodlot or membership in a community group that owns a woodlot and does not include casual access to woodlots owned by others. All analyses were conducted in R (R Core Team 2016) and Generalized linear mixed-effects models were fit in package lme4 (Bates et al. 2019). Top models presented here were selected based on AIC in generalized models (Pan 2001). The log-linear model was fit using package MASS (Venables and Ripley 2002).

## Results

Across the study area, households varied in their livelihood strategies, demographics, physical resources, farming practices, wood use, and stretcher group characteristics (Table 1).

**Table 1:** Values of variables generated via household surveys in the study regions. Values are displayed as regional averages, modes, and proportions as appropriate (with standard deviation reported in parentheses as appropriate)

	Variable	Mean (SD)/most common response
Demographics	Household size	5.9 (2.2)
	Household education^a^	2.2 (0.8)
	Households with off-farm employment	21%
Physical Assets	Primary water source	River
	Toilet type	Pit latrine (no slab)
	Households that own a radio	64%
Financial Assets	Poverty probability^b^	28.3% (11.5)
Farming	Land owned (hectares)	2.5 (6.8)
	Years land owned	23.7 (16.6)
	Primary crop	Beans
	Secondary crop	Sweet potatoes
	Land planted with trees (hectares)	1.1 (4.6)
	Households using fertilizer	15%
	Soil quality^c^	2.9 (1.0)
	Livestock per household	3.9 (5.7)
Health and well-being	Household health^d^	1.8 (0.4)
	Months of food insecurity	2.3 (2.4)
Wood resources	Primary fuel source	Firewood
	Total types of wood products used	3.7 (1.1)
	Total types of wood products sold	1.2 (1.4)
	Households able to meet wood product needs	54%
Woodlot	Woodlot ownership	84%
	Woodlot size (hectares)	0.30 (0.35)
	Years woodlot ownership	14.8 (10.9)
	Primary woodlot species planted	Eucalyptus
	Access to private woodlot only	60%
	Access to group-owned woodlot only	4%
	Access to both private and group woodlot	24%
	No woodlot access	11%
Stretcher groups	Stretcher group membership	97%
	Stretcher group size	98.3 (61.8)
	Years of membership	19.4 (12.7)

^a^Measured as average completed education index (where 1= no education, 2 = Primary school, 3 = junior secondary school/form 1–4) for all household adults 18 years old and above

^b^Poverty Probability is defined according to the Uganda Poverty Probability index (PPI) based on household size, school enrollment, literacy, dwelling construction materials, and asset ownership (Schreiner 2011). The value represents the average percent likelihood that a household is below the national poverty line

^c^Based on respondent report and scaled where 1 = very poor, 2 = poor, 3 = ok, 4 = good, 5 = very good

^d^Based on respondent report for all household members where 1 indicates unhealthy and 3 indicates very healthy

### Reliance on woodlots and wood products

In addition to the 99% percent of households that relied on firewood as their primary source of energy, 25% of households used charcoal as a non-primary energy source. Other important locally sourced wood products included crop stakes (used by 96% of households), building poles (used by 83% of households), and timber (used by 58% of households). Fifty-six percent of households also sold wood products, primarily timber (32% of households), building poles (24%), charcoal (15%), stakes (13%), and firewood (9%). Seventy-six percent of households sourced firewood from their own land. This included privately owned farmland and privately owned woodlots. Twenty-one percent of households sourced firewood from another person’s privately owned land for free. The remaining households sourced firewood from community-owned forests (1%), purchased it from others (1%), or sourced it from other locations (1%).

Despite access to private forests and woodlots, many households (40%) reported being unable to meet their wood product needs and most households (80%) reported experiencing difficulty meeting their wood product needs. Households cited land scarcity and cost as a primary barrier to meeting wood product needs. Households varied in their ability to meet household wood needs, ranging from 45 to 90% of households across villages.

### Woodlot ownership

**Fig. 3:**
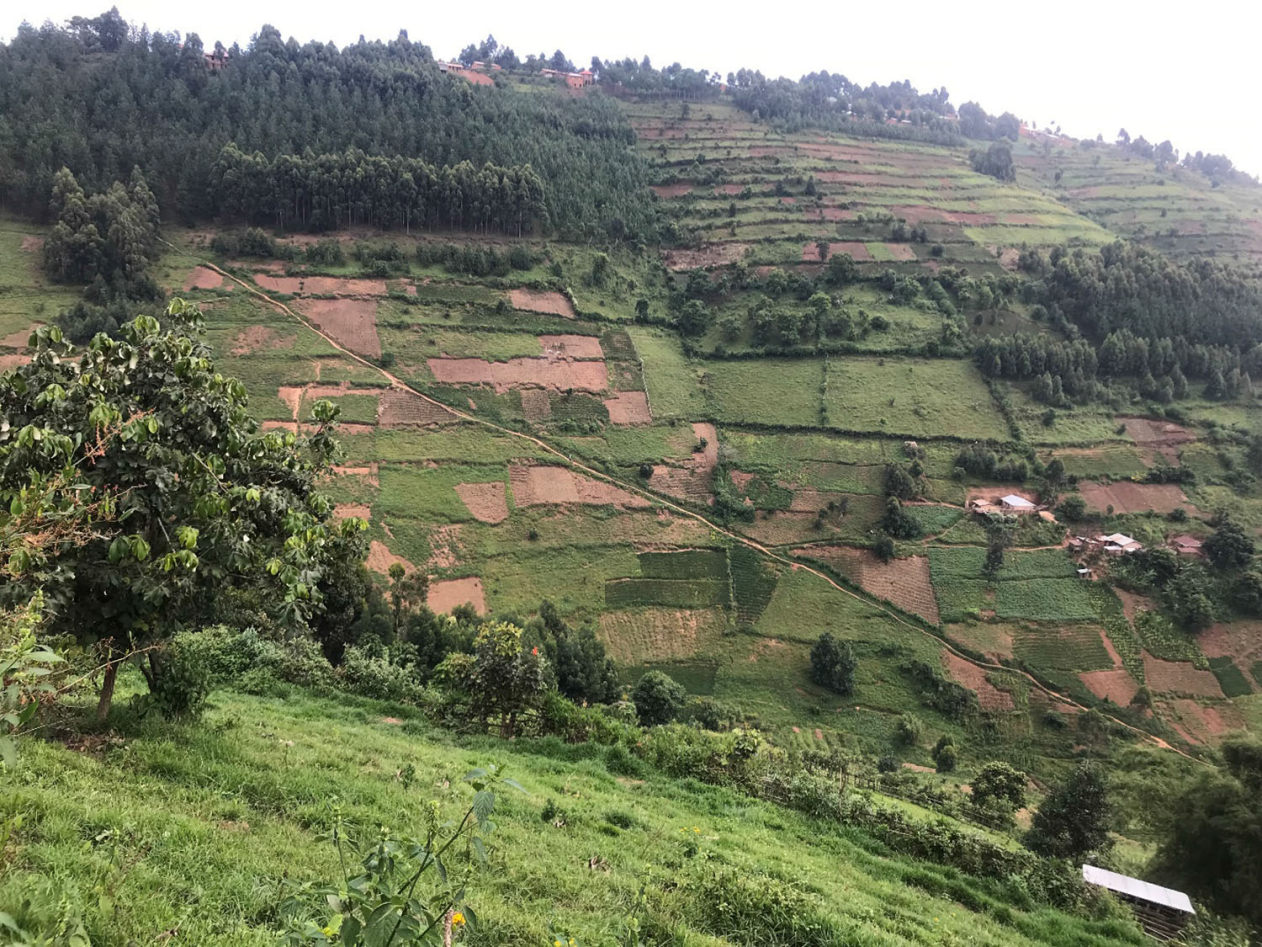
Privately owned pine and eucalyptus woodlots in communities outside Bwindi Impenetrable National Park. Photo by Joel Hartter

Across the study area, private woodlot ownership was high: eighty-five percent of households owned a private woodlot. Privately owned woodlots ranged in size from 0.04 hectares to 3.2 hectares (0.3 ± 0.4 ha) and households had owned them for between 1 and 56 years (13.7 ± 9.6 years). Most commonly, woodlots species were a mixture of eucalyptus (*Eucalyptus* spp., present in 93% of woodlots, Fig. 3) and pine (*Pinus patula*) (present in 13% of woodlots), with the two species occasionally mixed together in one woodlot. Woodlot ownership was positively associated with maximum household education, household size, having off-farm employment in the household, the total amount of land owned by a household, and belonging to a stretcher group (Table 2).

**Table 2:** Results from a logistic regression with woodlot ownership as the response variable (*n* = 396). Estimates report the influence of wealth, maximum household education, employment, land ownership, and stretcher group membership on the probability of private woodlot ownership

Model variable	Estimate	95% CI	SE	Odds ratio	*P*
(Intercept)	− 2.04	[− 4.19, 0.18]	1.10	0.13	0.11
Poverty probability	− 0.02	[− 1.04, 0.30]	0.02	0.98	0.30
Maximum household education	0.62	[0.20, 1.04]	0.22	1.86	< 0.01
Household employment	0.17	[0. − 0.57, 0.92]	0.38	1.19	0.65
Amount of land owned	0.66	[0.38, 0.95]	0.14	1.94	< 0.01
Stretcher group membership	1.18	[0.33, 2.69]	0.77	3.25	0.03

Households that owned a woodlot were more likely to report that they could meet their household wood product needs than households that did not own a woodlot (Table 3). About 75% of households that owned woodlots reported being able to meet their needs, compared to ~55% of those that did not own a woodlot. Additionally, woodlot size and duration of woodlot ownership were positively associated with the likelihood of a household meeting its wood product needs (Table 3).

**Table 3:** Results from a logistic regression with household ability to meet wood product needs as the response variable (*n* = 410). Estimates report the influence of woodlot ownership, woodlot size, and duration of woodlot ownership on the probability of meeting household wood needs along with model covariates

Model variable	Estimate	95% CI	SE	Odds ratio	*P*
Woodlot ownership	0.79	[0.16, 1.43]	0.32	2.21	0.01
Woodlot size	1.13	[0.53, 1.83]	0.33	3.10	< 0.01
Duration of woodlot ownership	0.04	[0.02, 0.07]	0.01	1.04	< 0.01
Household employment	0.95	[0.38, 1.55]	0.30	2.60	< 0.01
Poverty probability	0.002	[− 0.03, 0.04]	0.02	1.00	0.91
Maximum household education	− 0.09	[− 0.33, 0.16]	0.12	0.92	0.47
Amount of land owned	0.05	[0.02, 0.08]	0.015	1.05	< 0.01

### Woodlot management

Households used and managed woodlots in a variety of ways. Many woodlot owners reported collecting and allowing collection by others of downed branches within woodlots. Generally, the most lucrative use of woodlots was through harvest of poles and timber to sell, both within and beyond the village. Depending on the primary species grown in the woodlot, farmers reported intercropping; where eucalyptus is grown, this was challenging, where pine is grown, respondents reported that farming was more manageable. Consistently, households reported that private woodlots were insufficient for daily household wood product needs. Despite this, very few respondents (< 1%) reported purchasing wood products. Some households also owned forests with native species, and reported that they often persisted due to the efforts of conservation organizations and other NGOs.

### Collective action and woodlot management

Household survey data demonstrated the role of stretcher groups in collective action and governance in our study communities. The primary organizations that owned and managed collectively owned forests and woodlots were stretcher groups. Ninety-eight percent of survey respondents reported being a member of a stretcher group. Stretcher group membership ranged from 4 to 450 (69 ± 53 members). Membership in stretcher groups required attendance at regular meetings, payment of monthly dues of typically 1 000 Ugandan shillings (~ US $0.30), and engagement in a range of activities (e.g., transporting the ill, voting, land maintenance).

Data from focus group interviews revealed practices associated with the governance, benefits, and conflict management within stretcher groups. Stretcher groups had an elected executive committee with set term limits and explicit rules and regulations governing participation. Respondents reported that the benefits of participation in stretcher groups included access to land, social networks, economic support during times of stress, income from stretcher group activities, loans, and support for health care and funeral expenses. Eighty percent of stretcher groups owned one or more woodlots. Woodlots were used for collection of branches for firewood and timber and pole harvest for construction (e.g., tables, building poles, coffins). As with privately owned woodlots, the primary tree species were pine and eucalyptus. Privately owned woodlots and group-owned woodlots are of similar acreage. Privately owned lots are, on average, 0.30 hectares, while group woodlots are 0.28 hectares on average.

While there was variation in wood product use across households with access to different types of woodlots, access to group-owned woodlots did not correlate with ability to meet household wood product needs (Table 4). The *χ*^2^ test indicated that there is an association between access type and ability to meet wood product needs. The best fitting log-linear model indicated an interaction between access type and meeting wood product needs, with households with access to private woodlots having two times estimated odds of meeting wood product needs than those with access to group lots. Less than 1% of households that only had access to group woodlots sold wood products compared to 62% of households with access to both group-owned and private woodlots. Additionally, of those households that sold wood products, 87% sourced those wood products from private woodlots and only 2% sourced from group-owned woodlots.

**Table 4:** Contingency table displaying woodlot access type (access to both private and group-owned woodlots, access to only private woodlots, access to only group-owned woodlots, and access to no woodlots) and ability to meet household wood product needs (Pearson *χ*^2^: 8.99, df = 3, *p* = 0.029). Expected values are shown in parentheses

Woodlot access type	Met wood product needs	
	No	Yes
Access to group lots only	7 (5)	9 (11)
Access to private woodlots only	65 (72)	182 (175)
Access to both private and group-owned woodlots	26 (29)	72 (69)
No woodlot access	21 (13)	25 (33)

## Discussion

### Smallholder reliance on wood products sourced from private woodlots

For many of the most vulnerable global populations, wood products are critical to livelihood activities and well-being (Shackleton and Shackleton 2004; Angelsen et al. 2014). Our study demonstrated that the wood product needs of farming communities in a densely populated tropical forest and agricultural landscape were supported by access to woodlots. Nearly all households relied heavily on firewood, timber, and other wood products for daily livelihood activities. Woodlot ownership was associated with higher asset levels (e.g., reduced poverty probability, education, employment) and woodlot ownership supported access to and sale of various wood products. While our results point to a relationship between household assets and woodlot ownership, we do not have the data to demonstrate directionality or causality in this relationship, and other work in the region identifies wealth and access to resources as factors driving woodlot ownership (Naughton-Treves et al. 2011). Despite this, our results demonstrate the value of woodlots for wood product access and livelihoods in the region.

### Woodlot management strategies and livelihoods

In many contexts, collective action and collective governance of forests and forest resources are important for meeting household needs and coping with stress while minimizing environmental degradation (Maskey et al. 2006; Weyerhaeuser et al. 2006). Strong social networks and locally supported governance structures provide promising opportunities for natural resource management and livelihood resilience. In southwestern Uganda, stretcher groups provide such networks and governance institutions. Stretcher group members in our study area reported increased access to shared woodlot resources and to broader social networks and many positive benefits from stretcher group participation. Importantly, however, we did not find a relationship between access to collectively managed woodlot resources and reported ability to meet household woodlot needs. Households primarily sourced their wood products from privately owned woodlots (their own or others) and met other needs from collectively managed woodlots. For example, some respondents reported that collection of wood from others’ private land occasionally led to conflict, which was often mediated through local stretcher group mechanisms.

### Woodlots and collective management in tropical landscapes

When considering the future of tropical forest landscapes, a combination of privately owned and collectively owned woodlots and forests may benefit local communities in different ways, directly providing wood products and supporting social network engagement and collective action. In our study area, we saw this in action. Stretcher groups are resilient and trusted local institutions, which have emerged as important bodies to manage forests and woodlots for community use (Twongyirwe et al. 2011). However, woodlots are not unique in their role as social organizations. Community members reported that other community organizations, including churches, schools, and savings cooperative groups, also own and operate woodlots. Despite this, stretcher groups serve a unique role in that most households are members of a stretcher group and they operate as cohesive structures that are highly influential in the community. As a result, stretcher groups are likely better positioned than most local organizations to enforce bylaws made to protect woodlots and facilitate long-term woodlot management. Stretcher group-managed woodlots and forests remain intact outside BINP and serve as critical sources of wood products that the community can exploit during times of need. Importantly, however, the circumstances under which stretcher group-managed woodlot resources are utilized are fairly limited; they are primarily used for funerals, both for firewood and for coffin construction. Rarely are members granted permission to use for other reasons, including general home use. Stretcher groups are lasting community institutions that remain strong despite the various sociopolitical changes that have taken place in Uganda. Incorporating such locally relevant institutions while evaluating the potential for natural resources to meet existing community needs may provide a pathway for long-term collective natural resource management.

Along with stretcher group-owned woodlots, privately owned woodlots have emerged in this landscape as a critical source of wood products, supporting both household use and local incomes. Privately owned woodlots, planted with fast-growing, commercially valuable species remain the primary strategy for households to obtain wood products and increase income via wood sales. While our research did not explicitly address this, it is likely that these privately owned woodlots reduce encroachment into BINP for wood products and commercial activities, relative to a landscape where such woodlots were not created and maintained (Schönau 1991; Ham 2000). Our work highlights the important role of woodlots, and their various management and ownership strategies, as potential sources for wood products outside protected areas in a threatened landscape.

### The future of woodlots and livelihoods around BINP

The dense population of southwest Uganda is expected to grow and place increased pressure on already stressed agricultural systems (Pender et al. 2004). Further, Uganda is projected to become wetter under climate change (Diem et al. 2019; Salerno et al. 2019), increasing the risk of soil erosion and uncertainty in crop production (Karamage et al. 2017). We expect woodlots to continue to be important under changing environmental conditions and with population growth, potentially forcing households to choose between food crop production and woodlots. Woodlots around BINP potentially serve the multiple purposes of protection against soil erosion, supporting local institutions and social networks, and providing an income source separate from food crop production (Zoysa and Inoue 2016). Further, the increasing isolation of tropical African PAs increases the value of unprotected forests and woodlots, in part, for their ability to minimize pressure on remaining intact protected forests (Defries et al. 2005; Salerno et al. 2018). However, increasing food needs in the region are likely to limit the capacity of individuals to create new woodlots and an increase in woodlots may have positive impacts on carbon storage and soil retention, but potentially negative impacts on food security (Pender et al. 2004).

Given the success and prevalence of forestry in the region, there is also potential to develop agroforestry practices to balance tradeoffs between timber production and food needs. In other contexts, aside from pine and *eucalyptus* spp., several other tree species have been shown to support soil retention with minimal impacts on food crop growth (Siriri et al. 2013). Investing in long-term strategies that support agroforestry with a range of tree species may strengthen well-being outcomes and resilience to changing environmental conditions in the region.

In consideration of these tradeoffs and uncertainties regarding the long-term role and value of woodlots, we make the following recommendations. We recommend that the Ugandan government and conservation and development organizations working in this region should

- reduce dependency on fuelwood as a cooking fuel by supporting transitions to more sustainable fuel sources and efficient technologies (e.g., solar, improved cook stoves);
- support proactive regional land-use planning that acknowledges historic patterns and drivers of land conversion, anticipates future community land-use needs, and supports and empowers local communities to play a role in landscape management and development;
- develop strategies to effectively incorporate privately owned land and woodlots into regional planning for positive livelihood and conservation outcomes.

## Conclusion

Woodlots represent a critical resource for communities outside protected areas in southwest Uganda and in other densely populated tropical landscapes. They provide important energy sources, building materials, and sources of income. Household reliance on managed woodlots may support preservation of adjacent protected areas and maintain conservation goals in highly populated landscapes (Schönau 1991; Ham 2000). Privately owned woodlots provide the bulk of these wood products that households used consistently and heavily depended on. Collectively owned woodlots provide additional sources of wood products and shared income, and also provide social networks and collective governance structures to support livelihood resilience. Differences in woodlot management and community-level outcomes have long-term implications for both conservation and tropical forest sustainability and livelihood resilience. Long-term management of forests in these landscapes should consider the value of both privately owned and collectively owned woodlots and the mechanisms required to support them.
